# Effects of Night Shifts on Sleep, Cognitive Performance, and Anxiety in Emergency Nurses: An Observational Within‐Subject Study

**DOI:** 10.1155/jonm/1635579

**Published:** 2026-02-24

**Authors:** Carlos Casas-Méndez, Miguel Ángel Martín-Parrilla, Macarena C. Cáceres, Jesús Montanero-Fernández, Casimiro Fermín López-Jurado, Noelia Durán-Gómez

**Affiliations:** ^1^ Departamento de Enfermería, Facultad de Medicina y Ciencias de la Salud, Universidad de Extremadura, Badajoz, 06005, Spain, unex.es; ^2^ Departamento de Enfermería, Centro Universitario de Plasencia, Universidad de Extremadura, Plasencia, 10600, Spain, unex.es; ^3^ Grupo de Investigación Traslacional Biomédica y Sociosanitaria (CTS064), Instituto de Investigación Biosanitaria de Extremadura (INUBE), Badajoz, 06005, Spain; ^4^ Departamento de Matemáticas, Facultad de Medicina y Ciencias de la Salud, Universidad de Extremadura, Badajoz, 06005, Spain, unex.es

**Keywords:** anxiety, cerebral oxygenation, cognitive performance, emergency nurses, night shift work, prefrontal cortex, sleep duration

## Abstract

**Aims:**

To investigate the effects of night shifts (NS) on cerebral oxygenation, cognitive performance, and anxiety in emergency nurses and to examine the role of sleep duration in modulating these outcomes.

**Background:**

NS disrupt circadian rhythms, reduce sleep, and may impair prefrontal cortex function. Emergency nurses, who are exposed to high cognitive and emotional demands, are especially vulnerable to these disruptions, which may compromise psychological well‐being, occupational performance, and patient safety.

**Method:**

A prospective, observational, within‐subject, cross‐over study was conducted in 67 emergency nurses. Each participant was evaluated both after completing a NS and during a day shift following a full night of rest at home (NR), with the order of assessments randomly assigned and separated by a 14‐day washout period. Regional cerebral oxygenation (rSO_2_) and prefrontal cortex reactivity were measured using near‐infrared spectroscopy (NIRS) while performing a verbal fluency task. Cognitive performance, sleep disturbance, and state anxiety were assessed with validated instruments: State Trait Anxiety Inventory (STAI), Insomnia Severity Index, and NIRS with Verbal Fluency Test (VFT).

**Results:**

After NS, nurses showed a significant decline in rSO_2_ (67.65 ± 6.37 vs. 64.45 ± 6.78; *p* < 0.001), reduced performance in the VFT (47.19 ± 11.14 vs. 42.85 ± 11.99; *p* < 0.001), and increased state anxiety (14.63 ± 9.53 vs. 21.37 ± 7.67; *p* < 0.001). Shorter sleep duration during NS was associated with lower rSO_2_ mean in both conditions: NR and NS. Interestingly, nurses who reported longer sleep during NS exhibited higher rSO_2_ but paradoxically poorer cognitive performance, suggesting that those with shorter sleep may have engaged compensatory mechanisms in those with shorter sleep that allowed them to maintain task performance despite reduced rSO_2_.

**Conclusions:**

Reduced sleep during NS negatively affected cerebral oxygenation, cognitive performance, and emotional well‐being in emergency nurses. Longer self‐reported sleep did not necessarily confer cognitive benefits and may reflect inefficient or overestimated sleep.

**Implications for Nursing Management:**

Organizational strategies are needed to mitigate the adverse effects of NS work. Optimizing shift schedules, ensuring structured rest opportunities, and implementing programs aimed at reducing anxiety and supporting psychological well‐being could help protect nurses’ health, sustain performance, and improve patient safety.

## 1. Introduction

Nursing is a profession that entails a high level of responsibility in patient care, as it encompasses both health maintenance and the treatment of diseases [1]. To ensure continuity of care, nursing services are organized through shift work, which requires performing activities during periods normally reserved for rest. Because humans are biologically programmed to be active during the day and sleep at night, shift work disrupts circadian rhythms [2, 3], reducing total sleep time and increasing sleepiness during working hours [4].

These disruptions have been associated with higher levels of anxiety, fatigue, burnout, and insomnia, which not only affect nurses’ health but also compromise the quality of care [5]. Reynolds AC et al. reported that night shift (NS) workers with sleep disorders exhibit higher anxiety scores, indicating a deterioration in psychological well‐being [6]. From a management perspective, these consequences pose challenges for workforce sustainability, increase the likelihood of errors, and affect patient safety, particularly in high‐complexity settings where nurses perform critical tasks. For instance, medication administration accounts for approximately 40% of clinical activities, positioning nursing staff as key agents in preventing errors, especially in intensive care units or emergency departments [7].

At the neurophysiological level, prolonged exposure to shift work may affect the functioning of the prefrontal cortex (PFC), a region supporting essential cognitive functions such as working memory, attention, and decision‐making [8, 9]. The PFC is also highly sensitive to sleep loss: its activity declines during the transition to sleep and is further impaired under sleep deprivation [10, 11]. Neuroimaging studies using near‐infrared spectroscopy (NIRS) have shown reduced prefrontal activation in individuals with insomnia during cognitive tasks such as verbal fluency, reflecting altered cerebral hemodynamic [12–15]. Likewise, reduced functional connectivity in the PFC has been observed in association with greater insomnia severity [16].

In the context of nursing practice, Durán‐Gómez et al. demonstrated that sleep deprivation associated with night work leads to lower dorsolateral PFC reactivity and poorer cognitive performance, indicating that stress, fatigue, and sleep loss significantly alter nurses’ brain activity compared with conditions of adequate rest [17].

From a healthcare management standpoint, the effects of night work on staff sleep, cognitive function, and psychological health constitute a significant challenge. Decreased performance, an increased risk of errors, and a deterioration in occupational well‐being directly affect care quality, patient safety, and organizational efficiency. Understanding these neurophysiological and behavioral processes provides a basis for management decisions aimed at optimizing shift scheduling, improving working conditions, reducing costs associated with absenteeism, and increasing the operational capacity of high‐demand services such as emergency departments.

The objectives of this study were to examine the effects of NS work on cerebral oxygenation, cognitive performance, and state anxiety in emergency nursing staff, and to explore how these outcomes vary according to self‐reported sleep obtained during NS. Beyond the physiological and cognitive dimensions, this study sought to situate these findings within the organizational context of emergency care, characterized by high workload, time pressure, and staff turnover.

## 2. Methods

### 2.1. Research Design

A prospective, observational, within‐subject, cross‐over study was conducted and reported in accordance with the STROBE checklist [18]. Each participant served as their own control and was evaluated under both conditions: NS and NR.

The assessments followed the following schedule: the NS condition was conducted between 7:00 a.m. and 7:45 a.m., that is, during the final 45 min of the NS (10:00 p.m.–8:00 a.m.). This timing was established to ensure that measurements were taken at the end of the shift without interfering with participants’ personal or family responsibilities after completing their workday.

The NR condition was subsequently conducted between 2:15 p.m. and 3:00 p.m., immediately before the start of the afternoon shift (3:00 p.m.–10:00 p.m.). This procedure ensured that assessments did not interfere with participants’ work performance while also providing a consistent evaluation time free from fatigue associated with clinical duties.

During the 14‐day washout period, it was not possible to fully control the distribution of NS assigned by the unit’s scheduling system. Completing more than one NS within this interval depended on staff availability, individual preferences and decisions, and operational needs of the service—factors beyond the control of the study. Nevertheless, the NS condition was scheduled to occur after the NS within the afternoon–morning/night or afternoon–afternoon/night work cycles. Conversely, the NR condition was scheduled 72 h after the last NS to avoid residual effects of sleep deprivation, ensuring that participants did not work any shifts during that interval. This period coincided with the two rest days routinely assigned following a NS.

This design allowed for direct within‐subject comparisons reducing interindividual variability and thereby enhancing methodological robustness.

### 2.2. Study Population

The study included Spanish nurses and nursing assistants (*N* = 67) who work in the Hospital Emergency Service (HES) of the University Hospital of Badajoz. The HES where the study was conducted is functionally organized into four main clinical areas: triage, general consultation rooms, trauma care, and an observation unit. Nursing staff may be assigned to different areas during their shifts depending on patient flow, clinical demand, and staffing requirements. Both professional profiles were included because the study focused on the effects of sleep restriction on cognitive performance and functional readiness during emergency care, rather than on differences in clinical responsibility or decision‐making. From a patient safety and management perspective, reduced performance due to fatigue may have relevant consequences regardless of professional role in this high‐demand setting.

The sample was collected between 2020 and 2024. Individuals with pre‐existing sleep disorders, significant comorbidities, or psychoactive medication use were excluded to avoid confounding the assessment of anxiety, sleep quality, or cognitive performance. Staff who did not participate in at least three NS over a 30‐day period were also excluded. Participants worked under a rotating shift system consisting of morning shifts (8:00 a.m.–3:00 p.m.), afternoon shifts (3:00 p.m.–10:00 p.m.), and NS (10:00 p.m.–8:00 a.m.), following combinations such as afternoon–morning/night or afternoon–afternoon/night, occasionally performing morning/night or afternoon/night shifts on the same day. Thus, sequences of afternoon–morning/night or afternoon–afternoon/night involved working 24 h across two consecutive days.

### 2.3. Data Collection Procedure

All data collection was conducted face‐to‐face in controlled hospital settings by a single trained researcher, blinded to the study hypotheses, thereby minimizing potential order and carryover effects. The researcher was a registered nurse with postgraduate training in health sciences and systematic instruction in the administration of psychometric and neurophysiological measures, which ensured methodological rigor. To reduce the risk of information bias, questionnaires were administered immediately after each condition to maximize recall accuracy, and standardized written instructions were provided to all participants. Data collection was performed in a quiet, private space within the hospital to guarantee confidentiality and minimize potential social desirability bias.

### 2.4. Instruments and Measures

Measurement bias was mitigated by using published methods and internationally validated instruments with high reliability. These measures collectively ensured that the study findings are robust, valid, and methodologically sound.

#### 2.4.1. Ad Hoc Sociodemographic Questionnaire

It consists of questions related to age, gender, marital status, time worked in the HES, time worked as a nurse or assistant, average number of NS worked per month, average time they can rest on an NS, have a child or family member in their care position, carrying out some technique or activity to reduce the stress caused by work and type of relaxation technique, need for a smoking break, drinking coffee or stimulating substances on the NS, being in accordance with the management of the unit, have enough time to do their job, if in a few years they see themselves working in the HES, and if they had intention to leave the unit.

#### 2.4.2. State Trait Anxiety Inventory (STAI)

The STAI [19] comprises 40 self‐report items designed to evaluate two aspects of anxiety: one’s typical tendency towards anxiety (Trait scale) and their current experience of anxiety symptoms (State scale). Respondents rate each item using a 4‐point Likert scale ranging from 1 to 4. The combined scores for both scales fall within the range of 20–80. Elevated total scores indicate greater severity of anxiety symptoms or a stronger predisposition toward anxiety. This instrument has been validated for use in Spanish‐speaking populations [20].

#### 2.4.3. Insomnia Severity Index (ISI)

The ISI [21] is a widely utilized questionnaire designed to assess the severity of insomnia symptoms in individuals. This instrument comprises seven self‐report items, each evaluating different dimensions of sleep difficulties. Respondents are required to rate the severity of their symptoms on a scale from 0 to 4, where 0 indicates no difficulty and 4 represents a very severe difficulty. The scores from each item are then summed to generate a total ISI score, ranging from 0 to 28. Higher scores on this scale indicate greater severity of insomnia symptoms. This tool has been adapted and validated for Spanish‐speaking populations [22].

#### 2.4.4. Verbal Fluency Task (VFT) and Procedure

A VFT was employed to test cognitive functions [23] while assessing PFC hemodynamic by NIRS. Tests of verbal fluency (VF) were performed on each subject in the two conditions: NR and NS. The VF evaluation was divided into two tests: (1) First, a verbal semantic fluency test, where the subject is asked to name all the elements within a given semantic category (animals, plants, and tools); (2) Second, a phonological VF test, in which the subject is asked to say all the words that begin with a particular syllable or letter (pa, la, ro, o, z). Each block lasted 60 s, with 20 s per category/syllable/letter and a 10‐s rest interval between blocks. In order to avoid the effect of memory, the semantic categories, syllables, and letters were modified in each condition. In the NR condition, we used three categories (animals, plants, and tools), two syllables (te, pa), and one letter (o). In the NS condition, subjects were asked to list food, sports, and films categories and words that start with/la/,/ro/and/z/. Incorrect responses included saying “pass”, listing peoples’ names, repeating words or producing grammatical variations of a previous word. Behavioral performance was assessed as the total number of correct words generated.

#### 2.4.5. NIRS and VFT

The INVOS 5100 Cerebral Oximeter, manufactured by Somanetics Corporation in Troy, MI, USA, was employed to noninvasively assess rSO_2_ in both hemispheres of the PFC. NIRS was utilized to quantify concentrations of [oxy‐Hb]) and [deoxy‐Hb]. The calculation of near‐infrared light absorption by [oxy‐Hb] and [deoxy‐Hb] was executed using a modified Beer–Lambert law [24]. The relative amounts of both are used to calculate rSO_2_ and their cortical concentration changes are used as an indirect indicator of regional brain activation. The relationship between a decrease in [deoxy‐Hb] (and consequently an increase in rSO_2_) and an increase in the blood‐oxygenation‐level‐dependent signal of NIRS is a measure of cerebral activation. rSO_2_ was calculated assuming an arterial‐to‐venous blood ratio of 25:75%. The INVOS provides real‐time measurement and a display of rSO_2_ in the microvasculature beneath the sensor. The two disposable LED sensors alternated between emitting 710 and 830 nm wavelengths of light, absorbed by hemoglobin. The two receiving optodes were 3 and 4 cm in distance from the LED. Light travels from the sensor’s light‐emitting diode to either a proximal or distal detector, permitting separate data processing of shallow and deep optical signals.

Before the beginning of the task, participants were instrumented with sensors for the left and right frontal lobes at the dorsolateral level of the PFC. The sensors were securely affixed to the scalp. rSO2 was measured while the participant performed a word fluency task under the following conditions: (1) rest (pre‐test baseline, 1 min); (2) verbal fluency test (2 min); (3) rest (post‐task baseline, 1 min) in NR and NS conditions. The measurements obtained were rSO_2_‐NR_1_, rSO_2_‐NR_2_, and rSO_2_‐NR_3_ for the NR condition, and rSO_2_‐NS_1_, rSO_2_‐NS_2_, and rSO_2_‐NS_3_ for the NS condition, recorded in both hemispheres. The mean was calculated from the values recorded by two channels in the dorsolateral area of the PFC, yielding average rSO_2_‐NR and average rSO_2_‐NS. Throughout this period, the subject sat on a comfortable chair in a room that was illuminated by daylight. The sitting position was required to ensure comparability across studies because of spontaneous physiological oscillations, which could influence the NIRS signal quality and are posture dependent [25].

### 2.5. Statistical Analysis

The sample size was *N* = 67. The minimum sample size required to ensure sufficient power (at least 0.90), with a significance level of 0.05, to detect a moderate effect size (*δ* ≤ 0.500) in paired *t*‐tests is *N* = 44. Furthermore, a sample size greater than *N* = 30 is usually sufficient to compensate for a moderate violation of the normality assumption. Therefore, our sample size was sufficient in both respects.

Descriptive statistics were expressed as mean ± standard deviation, percentages, and Pearson’s correlation coefficients. In the statistical inference phase, given the large number of rSO_2_ measurements, the risk of type I errors was addressed by condensing the 12 original rSO_2_ measurements into two composite variables: rSO_2_‐NR and rSO_2_‐NS, calculated as the average of all values recorded in each condition. A preliminary principal component analysis (PCA) supported the validity of this data reduction strategy.

All primary outcomes (ISI, rSO_2_‐NR, rSO_2_‐NS, STAI‐NR, STAI‐NS, VFT‐NR, and VFT‐NS) showed acceptable adherence to a normal distribution or moderate skewness. Based on these results, parametric tests were applied. Specifically, paired *t*‐tests were used to compare day and night results, Pearson’s correlation coefficients to examine relationships between main variables, and one‐way ANOVA to compare groups. Nonparametric alternatives (Wilcoxon, Spearman, and Kruskal–Wallis tests) were also applied to confirm the results.

### 2.6. Ethical Considerations

The study was conducted in accordance with the Declaration of Helsinki and Spanish Organic Law 3/2018 on the Protection of Personal Data and Digital Rights. The Bioethics and Biosafety Commission of the University of Extremadura confirmed that the study complied with essential ethical standards (reg. number: 204//2023). Informed consent was obtained from all participants, who were provided with comprehensive information regarding the study’s aims, procedures, and potential risks, ensuring that their participation was voluntary and that they could withdraw at any stage without any negative consequences.

## 3. Results

A descriptive analysis of the general variables is shown in Table 1, distinguishing between primary and secondary outcomes. ISI, rSO_2_‐NR, STAI‐NS, VFT‐NR, and VFT‐NS showed a good fit to a normal distribution according to Shapiro–Wilk’s test (*p* = 0.139, *p* = 0.526, *p* = 0.184, *p* = 0.282, and *p* = 0.071, respectively), while rSO_2_‐NS and STAI‐NR and fitted worse (*p* = 0.001, *p* < 0.001) with moderate skewness (*g*_1_ = −1.088 and *g*_1_ = 1.374, respectively).

**TABLE 1:** Descriptive of primary and secondary outcomes.

	*N* = 67		Mean ± S.D.	Frequency (%)
Primary	ISI		8.49 ± 4.11	
	rSO2‐NR		67.65 ± 6.37	
	rSO2‐NS		64.45 ± 6.78	
	State Anxiety‐NR		14.63 ± 9.53	
	State Anxiety‐NS		21.37 ± 7.67	
	VFT‐NR		47.19 ± 11.14	
	VFT‐NS		42.85 ± 11.99	

Secondary	Age		40.15 ± 8.40	
	Duration in Emergency Department (months)		57.06 ± 62.65	
	Duration in any Department (months)		171.57 ± 90.90	
	Gender	Female		64 (95.5%)
		Male		3 (4.5%)
	NS reported sleep	0′‐30′		23 (34.3%)
		1–1 h 30′		29 (43.3%)
		2–3 h		15 (22.4%)
	Family responsibilities	yes		34 (50.7%)
		no		33 (49.3%)
	Smoker	yes		9 (13.4%)
		no		58 (86.6%)
	Engaged in relaxation techniques	yes		34 (50.7%)
		no		33 (49.3%)
	Consumer of coffee	yes		39 (58.2%)
		no		28 (41.8%)
	Satisfied with management of the unity	yes		20 (29.9%)
		no		47 (70.1%)
	Have sufficient time to complete properly the tasks	yes		10 (14.9%)
		no		57 (85.1%)
	Would remain in the current service in the future	yes		23 (34.3%)
		no		44 (65.7%)
	Would leave immediately if given the opportunity	yes		36 (53.7%)
		no		31 (46.3%)

The correlations between the primary outcomes are shown in Table 2. None of the secondary, except the number of hours slept during NS, demonstrated a correlation with the primary outcomes. As a result, this variable was included in the subsequent inference analysis.

**TABLE 2:** Pearson’s *r* correlation coefficient between the primary outcomes.

*N* = 67	Average rSO_2_‐NR	Average rSO_2_‐NS	State Anxiety‐NR	State Anxiety‐NS	VFT‐NR	VFT‐NS
ISI	−0.197	−0.167	0.232	0.275^∗^	0.191	0.087
rSO_2_‐NR		0.680^∗∗^	0.022	−0.149	−0.116	0.020
rSO_2_‐NS			−0.0091	−0.125	−0.229	−0.208
State Anxiety‐NR				0.353^∗∗^	0.187	0.234
State Anxiety‐NS					0.035	−0.022
VFT‐NR						0.775^∗∗^

^∗^Correlation is significant at the level 0.05.

^∗∗^Correlation is significant at the level 0.01.

**FIGURE 1:**
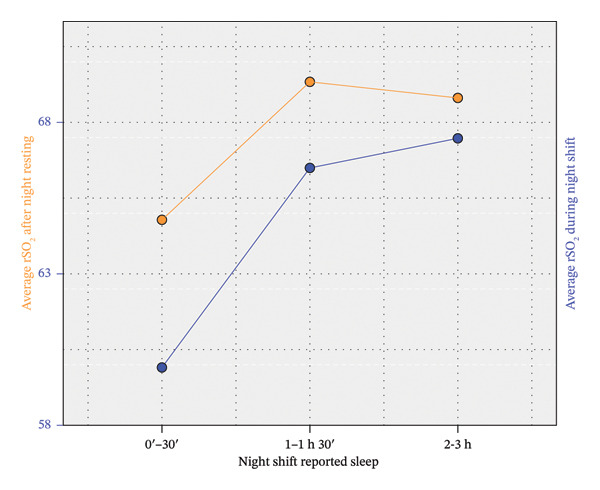
Differences in rSO_2_ scores by shift (NS vs. NR) and self‐reported sleep duration.

**FIGURE 2:**
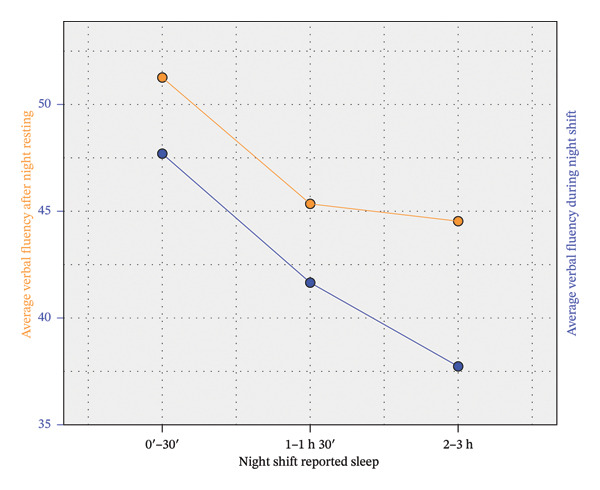
Differences in VFT scores by shift (NS vs. NR) and self‐reported sleep duration.

Table 2 shows direct correlations between NR and NS conditions for rSO_2_, state anxiety, and VFT, and a significant association between insomnia and state anxiety‐NS. The lack of correlation between rSO_2_ and the rest of variables can be partially explained if we split the sample into three groups according to the number of hours slept during NS. It is summarized in Table 3, which shows the difference in the primary outcomes between three groups. A one‐way ANOVA followed by Bonferroni’s post hoc method was applied to compare their means. The average rSO_2_‐NS for the group of nurses who slept for a maximum of 30 min stands out in Table 3, as it is the only one that differs significantly from the rest after applying a conservative Bonferroni correction. Thus, this group showed a particularly poor rSO_2_ level during the NS (see Figure 1). However, these individuals obtained significantly better results in VFT‐NS than the group of nurses who slept between 2 and 3 h (see Figure 2). Table 3 also shows an increase in anxiety and a decrease in rSO_2_ and VFT from the NR condition to the NS condition. This is analyzed in more detail in Table 4.

**TABLE 3:** Mean ± SD of the primary outcomes categorized by sleep duration during NS, analyzed using one‐way ANOVA and Bonferroni post hoc tests.

	0–30 min (*N* = 23)	1–1 h 30 min (*N* = 29)	2–3 h (*N* = 15)	ANOVA
ISI	8.07^a^ ± 3.96	8.97^a^ ± 3.98	8.20^a^ ± 4.76	*η* ^2^ = 0.011, *p* = 0.709
rSO_2_‐NR	64.78^a^ ± 7.37	69.34^b^ ± 5.79	68.8^a,b^ ± 4.20	*η* ^2^ = 0.109, *p* = 0.025
rSO_2_‐NS	59.91^a^ ± 7.78	66.50^b^ ± 4.77	67.47^b^ ± 4.86	*η* ^2^ = 0.242, *p* < 0.001
State Anxiety‐NR	18.35^a^ ± 9.59	12.69^a^ ± 9.30	12.67^a^ ± 8.71	*η* ^2^ = 0.081, *p* = 0.067
State Anxiety‐NS	23.74^a^ ± 8.55	19.93^a^ ± 6.34	20.53^a^ ± 8.24	*η* ^2^ = 0.051, *p* = 0.185
VFT‐NR	51.26^a^ ± 8.99	45.34^a^ ± 11.10	44.53^a^ ± 13.00	*η* ^2^ = 0.072, *p* = 0.093
VFT‐NS	47.70^b^ ± 13.41	41.66^a,b^ ± 10.48	37.73^a^ ± 10.30	*η* ^2^ = 0.103, *p* = 0.031

*Note:* In the table, a significant difference between the two groups according to the Bonferroni post hoc test is indicated only when they do not share any letters (^a,b^) in common. The size of the effect *η* ^2^ is also indicated.

**Table 4:** Mean an IC 95% for the difference between the NR and the NS conditions, categorized by number of hours slept during NS and overall.

Difference NR‐NS	Average difference (CI 95%)			
	Groups by number of hours slept during NS			
	0–30 min (*N* = 23)	1–1 h 30 min (*N* = 29)	2–3 h (*N* = 15)	Overall (*N* = 67)
rSO_2_	4.87 (2.07, 7.68)	2.84 (0.91, 4.77)	1.33 (1.12, 2.55)	3.20 (1.92, 4.49)
State Anxiety	−5.39 (−8.75, −2.03)	−7.24 (−11.52, −2.96)	−7.87 (−13.66, −2.07)	−6.74 (−9.16, −4.33)
VFT	3.56 (−0.52, 7.65)	3.69 (0.95, 6.43)	6.80 (3.58, 10.02)	4.34 (2.44, 6.24)

Table 4 shows the means and 95% CI of the differences between the NR and NS conditions for these variables, both separately according to the number of hours slept and overall. The difference between the three groups was tested by one‐way ANOVA without any significant difference. Namely, it was obtained *p* = 0.113 for rSO_2_, *p* = 0.713 for state anxiety, and *p* = 0.388 for VFT. In other words, the negative impact of sleep restriction did not depend clearly on participants’ habitual sleep duration. Next, the overall difference between the NR and NS conditions was tested using the paired *t*‐test, with significant results (*p* < 0.001 and Cohen’s *d* = 0.608 for rSO_2_, *p* < 0.001 and *d* = 681 for anxiety and *p* < 0.001 and *d* = 0.557 for VFT).

**FIGURE 3:**
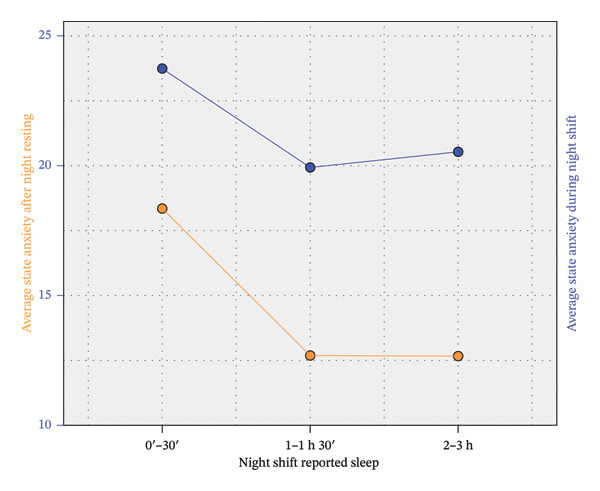
Differences in anxiety scores by shift (NS vs. NR) and self‐reported sleep duration.

In summary, there was a significant positive correlation between NR and NS conditions for both rSO_2_, anxiety, and VFT, although a significant worsening is detected in NS for each variable. This worsening did not depend significantly on the number of hours slept during the NS, but rSO_2_ values were significantly lower in both the NR and NS conditions for nurses who slept for a maximum of 30 min. Conversely, VFT performance was worse in the NS condition for nurses who slept more than 2 h. These statements are illustrated in Figures 1, 2, and 3.

## 4. Discussion

The present study provides a multidimensional description of how NS work is associated with changes in cerebral oxygenation, cognitive performance, and state anxiety in emergency nursing staff, a professional group exposed to high cognitive, emotional, and organizational demands.

Across the sample, NS work was consistently associated with lower PFC oxygenation, poorer verbal fluency performance, and higher state anxiety compared with the NR condition. Although the cross‐over design does not allow causal inferences, the within‐subject comparison strengthens the interpretation that these changes are temporally linked to the NS condition rather than to stable individual characteristics. From a managerial perspective, this pattern suggests that NS may place staff in a physiological and cognitive state that is less optimal for tasks requiring executive functioning [26] and sustained attention [27]—core competencies in emergency care environments [28].

In parallel with these neurocognitive changes, a clear increase in state anxiety was observed during NS. This finding is particularly relevant in emergency nursing, where emotional regulation and interpersonal communication are critical for both patient safety and team functioning. Elevated anxiety during NS may further compromise cognitive efficiency, situational awareness, and risk appraisal, even in the absence of clinically significant anxiety disorders [28]. From an organizational and leadership perspective, this underscores that NS not only affect physiological and cognitive domains but also influence psychological states [29] that are directly relevant to performance, teamwork [30], and error risk [31].

A noteworthy contribution of this study is the nuanced role of sleep during NS. In routine clinical practice, emergency nursing staff may rest briefly during NS when workload and staffing levels allow and patient care remains fully covered, reflecting real‐world operational conditions rather than a formalized intervention. While overall deterioration from NR to NS was observed regardless of reported sleep duration, staff who slept a maximum of 30 min showed significantly lower cerebral oxygenation in both NR and NS conditions, whereas those who slept more than 2 hours during NS demonstrated poorer cognitive performance during the shift. These findings do not support a linear or uniformly protective effect of longer sleep opportunities during NS. Instead, they point to heterogeneous responses that may reflect differences in sleep fragmentation, timing of sleep episodes, or transient post‐sleep cognitive states. One possible interpretation is that poorer cognitive performance immediately following longer sleep episodes during NS may be influenced by sleep inertia, defined as a temporary reduction in alertness and cognitive efficiency following awakening [32]. Sleep inertia has been described in operational settings as particularly relevant when individuals are required to resume complex tasks shortly after waking [33]. In contrast, nursing staff who slept very little may have maintained task performance through increased cognitive effort or heightened arousal [34, 35], reflected in relatively better VFT despite lower cerebral oxygenation. Importantly, these interpretations are presented as plausible explanatory frameworks rather than definitive mechanisms, and alternative explanations—such as individual differences in fatigue tolerance or task engagement—cannot be ruled out.

From an organizational standpoint, these patterns have direct implications for how rest opportunities during NS are structured and managed. The findings suggest that simply allowing longer sleep periods during NS may not uniformly translate into better cognitive functioning at critical moments, particularly if staff are required to return immediately to high‐demand tasks. Nurse managers may therefore need to consider not only whether rest is possible, but also how rest periods are timed, how awakening is managed, and how task allocation is adjusted following sleep episodes to minimize potential performance decrements, as previous research on sleep inertia in operational settings suggests [36].

Beyond physiological and cognitive outcomes, the organizational context described by participants is highly informative for interpreting the results. A large proportion of the sample reported insufficient time to complete tasks, dissatisfaction with unit management, high turnover intention, and willingness to leave the service if given the opportunity. These indicators reflect a strained work environment that may amplify the effects of sleep restriction and anxiety observed during NS. While the present study did not model these organizational variables as predictors, their high prevalence underscores that cognitive and physiological strain occurs within a broader context of workload pressure and organizational stress [37].

Importantly, the findings of this study should be interpreted within the broader organizational context of rotating shift systems rather than attributing their effects exclusively to night work itself. Evidence from occupational health research suggests that frequent rotation of sleep‐wake schedules may be more detrimental than stable assignment to a fixed shift, even when that shift is nocturnal [38]. Constant disruption of circadian adaptation, combined with high clinical demands, may therefore represent a form of organizational strain that extends beyond individual tolerance to night work [39]. From this perspective, NS function not only as a temporal exposure but also as a manifestation of wider organizational practices that shape nurses’ physiological and cognitive load.

For nursing leadership, these findings reinforce the importance of addressing NS not only as an individual adaptation challenge but as a system‐level issue [40]. Staffing adequacy, realistic workload allocation, rotation planning, and recovery opportunities are managerial levers that may mitigate the cumulative impact of NS on staff functioning [41]. The observed increase in state anxiety during NS further highlights the need for supportive leadership practices, clear communication, and psychologically safe work environments, particularly during high‐demand shifts [42].

This study adds to the existing literature by integrating cerebral oxygenation data with cognitive performance and anxiety measures in an emergency nursing context, while also incorporating sleep duration during NS as a differentiating factor. Rather than demonstrating a simple benefit of longer sleep during NS, the findings reveal a more complex and operationally meaningful pattern that challenges assumptions about rest opportunities and performance. This perspective is especially valuable for nurse managers and health system leaders tasked with balancing staff well‐being, patient safety, and service continuity.

### 4.1. Strengths and Limitations

This study provides valuable insights into the cognitive and emotional consequences of NS work among emergency department personnel. A key strength lies in the combined use of objective cognitive performance measures and self‐reported emotional assessments, which together enable a comprehensive evaluation of the effects of sleep deprivation. The inclusion of a well‐defined sample working in a high‐stress clinical environment further enhances the generalizability of the findings to comparable healthcare settings. Importantly, the neurophysiological measures were related not only to the shift condition (NS vs. NR) but also to sleep duration during NS, a factor that has not been systematically integrated into previous analyses and thus represents a novel contribution.

Several limitations should also be acknowledged. The cross‐sectional design precludes the establishment of causal relationships between sleep duration and cognitive or emotional outcomes. Reliance on self‐reported sleep data introduces the risk of recall bias, and potentially relevant factors such as sleep quality, prior sleep debt, and sleep duration before the NR condition were not directly measured. Domestic care responsibilities were not taken into account and could have influenced the results. Additionally, we did not objectively assess whether participants’ habitual sleep duration fell within recommended ranges, and insomnia or chronically short sleep may have influenced NIRS responsiveness. We also did not objectively measure sleep inertia or circadian phase, which may affect post‐shift performance. Future studies employing longitudinal designs and objective sleep‐tracking methodologies are warranted to provide a more nuanced understanding of these associations.

## 5. Conclusion

This study highlights that NS work in emergency nursing is associated with decreased cerebral oxygenation, impaired cognitive performance, and increased state anxiety. While causality cannot be established, these within‐subject differences underscore night work as an organizational challenge affecting staff functioning and well‐being. Importantly, cognitive and emotional effects occurred in the context of high workload, low satisfaction with unit management, and limited time to complete tasks, emphasizing structural and managerial influences. Variability in performance according to sleep duration suggests that rest alone may not ensure readiness, pointing to the need for structured fatigue management strategies. These findings support the implementation of evidence‐informed leadership practices, optimized staffing, and scheduled rest periods to enhance nurse performance, safety, and retention during NS.

## 6. Implications for Nursing Management

The findings of this study provide actionable insights for nursing management in emergency care settings. First, the consistent decrease in cerebral oxygenation, cognitive performance, and anxiety during NS supports the need to reconsider highly rotating shift schedules. Where service organization allows, greater stability in shift assignment may reduce repeated circadian disruption and cumulative fatigue.

Second, the findings highlight that cognitive and emotional vulnerability during NS should be addressed primarily through organizational and workload‐related strategies rather than relying on informal or prolonged rest opportunities, which may not be feasible in emergency care settings. Given the unpredictability and high acuity of emergency departments, nursing management should focus on staffing adequacy, realistic task allocation, and minimizing unnecessary cognitive load during NS, particularly for safety‐critical activities. Aligning workload intensity with known periods of fatigue and ensuring sufficient human resources may represent more practical and sustainable approaches to mitigating performance decrements associated with night work.

Third, the high prevalence of insufficient time to complete tasks, dissatisfaction with unit management, and intention to leave the service observed in this sample indicates that the effects of night work occur within a broader context of organizational strain. Addressing staffing adequacy and workload distribution during NS is essential not only for protecting nurse well‐being but also for maintaining patient safety and workforce retention.

Finally, nursing management should recognize night work as a system‐level issue rather than an individual adaptation challenge. Policies that integrate fatigue risk management and equitable shift planning are likely to yield more sustainable outcomes for emergency services.

## Author Contributions

Conceptualization, C.C.‐M., M.Á.M.‐P., M.C.C., C.F.L.‐J., and N.D.‐G.; data curation, J.M.‐F.; formal analysis, J.M.‐F.; funding acquisition, N.D.‐G.; investigation, C.C.‐M. and M.Á.M.‐P.; methodology, C.C.‐M., M.Á.M.‐P., M.C.C., J.M.‐F., and N.D.‐G.; project administration, N.D.‐G.; resources, M.C.C. and N.D.‐G.; supervision, M.C.C. and N.D.‐G.; validation, M.C.C., M.Á.M.‐P., and N.D.‐G.; visualization, C.C.‐M., M.Á.M.‐P., M.C.C., J.M.‐F., and N.D.‐G.; writing–original draft, C.C.‐M., M.Á.M.‐P., M.C.C., and N.D.‐G.; writing–review and editing, C.C.‐M., M.Á.M.‐P., M.C.C., J.M.‐F., and N.D.‐G.

## Funding

This project has been co‐financed at 85% by the European Union (European Regional Development Fund) and the Regional Government of Extremadura. Managing Authority: Ministry of Finance. Reference number: GR24011.

## Disclosure

All authors have read and agreed to the published version of the manuscript.

## Conflicts of Interest

The authors declare no conflicts of interest.

## Data Availability

The data that support the findings of this study are available upon request from the corresponding author. The data are not publicly available due to ethical restrictions.
